# SOCIAL MEDIA AND SOCIAL WELL-BEING IN LATER LIFE

**DOI:** 10.1093/geroni/igz038.1197

**Published:** 2019-11-08

**Authors:** Kelly Quinn

**Affiliations:** University of Illinois at Chicago, Chicago, Illinois, United States

## Abstract

Social well-being is important to health, but maintaining social relations often becomes difficult in later life due to retirement, chronic disease, and the death of spouses and friends. Social media platforms, such as Facebook and Twitter, present accessible and low cost communication technologies that are associated with enhanced feelings of social connection and reduced loneliness in younger age groups. This paper examines whether similar benefits might arise for adults at older ages. Using a four-week social media training workshop as a randomized, controlled wait-list intervention, this study examines whether social benefits are realized among a group of novice social media users, aged 65+. Measures of social well-being, including social capital, loneliness, social connectedness, and social provisions, were assessed at pre- and multiple post-test intervals for differences related to social media learning. Findings revealed only small differences between groups in one dimension of social connectedness, that of social integration. As these findings seemingly contradict studies conducted with younger persons, the contexts of social media use in older adulthood are discussed. These include the relevance of lower social media adoption rates, as well as influences that intersect with an older person’s life stage, such as gaps in network coverage on technological platforms, perceptions of the value of weak connections, and a reduced digital skills base. These additional factors are relevant to understanding disparity in the benefits that can be obtained through the use of social media and highlight the differing needs that social media fulfill at varying life stages.

